# Real-World Experience with Targeted Therapy in BRAF Mutant Advanced Melanoma Patients: Results from a Multicenter Retrospective Observational Study Advanced Melanoma in Russia (Experience) (ADMIRE)

**DOI:** 10.3390/cancers13112529

**Published:** 2021-05-21

**Authors:** Kristina V. Orlova, Evgeniy V. Ledin, Natalia V. Zhukova, Rashida V. Orlova, Elena V. Karabina, Mikhail V. Volkonskiy, Daniil L. Stroyakovskiy, Aleksandr N. Yurchenkov, Svetlana A. Protsenko, Alexey V. Novik, Ludmila V. Vorotilina, Fedor V. Moiseenko, Victor L. Chang, Aleksandr I. Kazmin, Svetlana A. Tkachenko, Sergey V. Gamaunov, David R. Naskhletashvili, Igor V. Samoylenko, Anastasia S. Vikhrova, Igor A. Utyashev, Galina Yu. Kharkevich, Natalia N. Petenko, Irina Zh. Shubina, Lev V. Demidov

**Affiliations:** 1 FSBI “N.N. Blokhin National Medical Research Center of Oncology” of the Ministry of Health of the Russian Federation, 115478 Moscow, Russia; nas-david@yandex.ru (D.R.N.); igor.samoylenko@gmail.com (I.V.S.); semencova_a.s@mail.ru (A.S.V.); gkharkevich@mail.ru (G.Y.K.); n.petenko@gmail.com (N.N.P.); demidov.lev@gmail.com (L.V.D.); 2 Association Professional Melanoma Society (MELANOMA.PRO), 119192 Moscow, Russia; sdaniel@mail.ru (D.L.S.); s.protsenko@list.ru (S.A.P.); anovik@list.ru (A.V.N.); gamajnovs@mail.ru (S.V.G.); dr.utyashev@gmail.com (I.A.U.); 3 Clinical Hospital 2, MEDSI, 119071 Moscow, Russia; ledin@inbox.ru; 4 SBHI of Saint-Petersburg “City Clinical Oncology Dispensary”, 197022 Saint Petersburg, Russia; natalia-zhukova@yandex.ru (N.V.Z.); Orlova_rashida@mail.ru (R.V.O.); 5 St Petersburg University, 199034 Saint Petersburg, Russia; 6 SHI “Tula Region Oncology Dispensary”, 300040 Tula, Russia; kev-251@yandex.ru; 7 Moscow City Oncology Hospital No. 62, 143423 Moscow, Russia; mux19@yandex.ru (M.V.V.); dr.yurchenkov@gmail.com (A.N.Y.); 8 FSBI “N.N. Petrov National Medical Research Center of Oncology” of the Ministry of Health of the Russian Federation, 197758 Saint Petersburg, Russia; moiseenkofv@gmail.com; 9 BHI of Omsk Region “Clinical Oncology Dispensary”, 644046 Omsk, Russia; vorolu@rambler.ru; 10 SBHI “Saint-Petersburg Clinical Scientific and Practical Center for Specialized Types of Medical Care (Oncological)”, 197758 Saint Petersburg, Russia; 11 SBHI “Tambov Region Oncology Dispensary”, 392000 Tambov, Russia; ken_baxter@mail.ru; 12 BHI of Voronezh Region “Voronezh Region Clinical Oncology Dispensary”, 394036 Voronezh, Russia; kazmin13@mail.ru; 13 SBHI of Kaluga Region “Kaluga Region Clinical Oncology Dispensary”, 248007 Kaluga, Russia; sv0959@mail.ru; 14 Chuvash Autonomous Institution “Republic Clinical Oncology Dispensary” of Chuvash Republic MoH, 428020 Cheboksary, Russia; 15 Institute of Oncology, Hadassah Medical Moscow, 121205 Moscow, Russia

**Keywords:** BRAF, chart review, dabrafenib, vemurafenib, MEK, melanoma, trametinib, cobimetinib

## Abstract

**Simple Summary:**

Advanced melanoma is a highly aggressive disease with a poor prognosis. Recent clinical trials have shown that targeted therapy (TT) and immunotherapy (IT) lead to significant improvements in responses to treatment and the survival of advanced melanoma patients. However, little information is available in the form of real-world data on treatment patterns and clinical outcomes for patients with advanced BRAF V600 mutant melanoma. To approach this issue, we performed a retrospective study that involved 382 patients with advanced BRAF V600 mutant melanoma, who received TT in twelve medical centers. Our objectives were to evaluate clinical outcomes in real-world settings, as well as treatment patterns, adverse events, objective response rate (ORR), progression-free survival (PFS) and overall survival (OS). Considering these parameters, the results demonstrated the effectiveness of combined TT with BRAF plus MEK inhibitors in patients with brain metastases and across all lines of therapy, which was well-tolerated and manageable and showed a high safety profile.

**Abstract:**

Clinical trials of targeted therapy (TT) and immunotherapy (IT) for highly aggressive advanced melanoma have shown marked improvements in response and survival rates. However, real-world data on treatment patterns and clinical outcomes for patients with advanced BRAF V600 mutant melanoma are ultimately scarce. The study was designed as an observational retrospective chart review study, which included 382 patients with advanced BRAF V600 mutant melanoma, who received TT in a real-world setting and were not involved in clinical trials. The data were collected from twelve medical centers in Russia. The objective response rates (ORRs) to combined BRAFi plus MEKi and to BRAFi mono-therapy were 57.4% and 39.8%, respectively. The median progression-free survival (PFS) and median overall survival (OS) were 9.2 months and 22.6 months, respectively, for the combined first-line therapy; 9.4 months and 16.1 months, respectively, for the combined second-line therapy; and 7.4 months and 17.1 months, respectively, for the combined third- or higher-line therapy. Analysis of treatment patterns demonstrated the effectiveness of the combined TT with BRAF plus MEK inhibitors in patients with brain metastases, rare types of BRAF mutations, and across lines of therapy, as well as a well-tolerated and manageable safety profile.

## 1. Introduction

Historically, advanced melanoma was considered to be a chemotherapy-resistant malignancy with a poor prognosis and a median overall survival (mOS) of less than 12 months. Several combination chemotherapy regimens have been tested, but no survival benefits have been demonstrated [1,2,3,4,5,6]. Commonly used therapies have included cytotoxic chemotherapies and cytokine-based immunotherapy (interferon, interleukin-2), which were primarily palliative in nature, with no improvements in survival. In the last 10 years, new effective therapies have emerged which focus either on the BRAF V600E mutation (targeted BRAF-directed therapy) or against regulatory checkpoints (immunotherapy with checkpoint inhibitors). The advent of targeted therapy and checkpoint inhibitors has dramatically changed the landscape of treatment for metastatic melanoma. Rapid progress in BRAF and MEK targeted therapy (TT) and immunotherapy (IT) has led to significant improvements in objective response and survival rates in advanced melanoma patients [7,8,9,10,11,12,13,14,15].

Recently, a study of patients treated with BRAF-targeted therapy via dabrafenib plus trametinib in randomized phase 3 trials (COMBI-d and COMBI-v) has reported its results. This pooled 5-year analysis included a total of 563 patients who were randomized to receive dabrafenib plus trametinib (211 in the COMBI-d trial and 352 in the COMBI-v trial). The objective response rate (ORR) was 68% (383 of 563 patients), with 19% achieving a complete response. The 5-year progression-free survival (PFS) rate was 49% (95% CI, 39 to 58) among patients with a complete response. The PFS and overall survival (OS) rates were 19% (95% CI, 15 to 22) and 34% (95% CI, 30 to 38), respectively, at the 5-year end point for all patients included in the pooled analysis. The PFS and OS rates were higher in patients with normal lactate dehydrogenase (LDH) levels, at 25% (95% CI, 20 to 30) and 43% (95% CI, 38 to 49), respectively [8]. 

The COLUMBUS study evaluated the role of combination targeted therapy in the treatment of patients with BRAF+ advanced melanoma. In the phase 3, randomized trial in patients with BRAF-mutant melanoma, encorafenib plus binimetinib showed a favorable efficacy and safety profile compared with vemurafenib monotherapy. Progression-free survival in patients treated with encorafenib plus binimetinib was significantly longer than that in patients treated with vemurafenib, and progression-free survival results were consistent with those seen in previous trials with vemurafenib in that setting. The encorafenib plus binimetinib group had a median progression-free survival of 14.9 (95% CI, 11.0–18.5) months, whereas the encorafenib group had a median progression-free survival of 9.6 (7.5–14.8) months; (HR 0.75, 95% CI, 0.56–1.00; two-sided *p* = 0.051). More patients in the encorafenib plus binimetinib group attained an overall response compared with those in the encorafenib group (121 (63%; 95% CI, 56–70) vs. 98 (95% CI, 43–58)). These results confirm the higher efficacy of combination targeted therapy compared to BRAFi monotherapy [15].

BRAF inhibition in combination with MEK inhibition demonstrated improved efficacy compared to BRAF inhibition alone and led to the FDA approval of several BRAF/MEK inhibitors. These are dabrafenib/trametinib, vemurafenib/cobimetinib, and encorafenib/binimetinib. BRAF/MEK combined therapy has become a standard option for patients with BRAF V600-mutant melanoma.

The mitogen-activated protein kinase (MAPK) pathway is a critical proliferation pathway in melanoma. BRAF is a serine/threonine protein kinase activating the MAP kinase/ERK-signaling pathway. Approximately 50% of melanomas harbor activating BRAF mutations [16,17]. Among them, over 90% are at codon 600, and among these, over 90% are a single nucleotide mutation resulting in a substitution of glutamic acid for valine (BRAFV600E: nucleotide 1799 T >A; codon GTG >GAG).

Grant A. McArthur presented the 5-year results of another phase 3 trial (coBRIM) of combined BRAF-targeted therapy with vemurafenib and cobimetinib during the Society for Melanoma Research Congress at the end of 2019. The coBRIM study was a prospective, randomized, double-blind, phase III trial comparing vemurafenib (V) plus cobimetinib (C) and V plus placebo (NCT01689519). After a median follow-up of 14.2 months, the median PFS for V+C was 12.3 months (95% CI 9.5–13.4). A subgroup analysis for LDH levels at baseline also showed a decreased median PFS if patients had elevated LDH levels at baseline (8.2 months; 95% CI 7.3–10.6). Patients with normal LDH levels at baseline had a higher median PFS of 13.4 months (95% CI 12.0–16.5). The median follow-up for OS was 18.5 months, with a median OS of 22.3 months (95% CI 20.3–not estimable). The 2-year OS rate was 48.3% (95% CI 41.4–55.2). At the data cut-off, 70% (95% CI 63.5–75.3) of patients had an objective response, with 16% having a complete response (CR). In general, the median duration of response (DOR) was 13.0 months (95% CI 11.1–16.6) and 18.1 months (95% CI 14.8–not estimable) if the patient had a CR. The 5-year PFS and OS rates presented by Grant A. McArthur were 14% and 31%, respectively, for all patients in the combination group *(n =* 247) [18]. Efficacy results were published earlier by Ascierto et al. [19]. A pooled analysis of four randomized clinical trials (BRIM-2, NCT00949702; BRIM-3, NCT00949702; BRIM-7, NCT01271803; and coBRIM, NCT01689519) identified LDH levels and the sum of the longest diameters of target lesions (SLD) as significant baseline characteristics regarding survival outcomes in patients receiving V+C [20]. Patients with normal LDH levels and SLD  ≤  45 mm at baseline had a median OS of 27.2 months (95% CI 22.1–32.1) with V+C. In contrast, if patients had elevated LDH levels (≥ 2 × ULN), the median OS was 6.0 months (95% CI 5.3–6.8). The 3-year landmark OS was 53.3% (95% CI 39.5–67.1) and 8.8% (95% CI 0.0–18.4), respectively [20].

Another crucial characteristic regarding the survival outcome of melanoma patients is the presence of melanoma brain metastases. The abovementioned trials (COMBI-d/v, coBRIM) either excluded patients with a history of brain metastases or included them only if they were asymptomatic and had been previously treated. Regarding *BRAF* mutational status, all described trials (COMBI-d, COMBI-v, coBRIM) included patients harboring the *BRAF* V600E/K mutation only, although in clinical practice patients with less frequent *BRAF* mutations are registered as well.

Although there is comprehensive and increasing evidence about the clinical efficacy and outcomes of randomized controlled trials in patients with advanced BRAF mutant melanoma, real-word data on treatment patterns and clinical outcomes are rather scarce. The “real-world” patient populations of metastatic melanoma are inadequately represented in the investigations of clinical trials. Real-word evidence following the randomized trials should better define the effectiveness of treatments in clinical practice, including subgroups of patients who are usually excluded or under-represented in the trials, and may provide clinically-rich insights into what actually happens in everyday practice and the reasons for the discrepancies between the expected and achieved results [21]. Therefore, real world data are essentially important since they complement the results obtained from randomized controlled trials [22].

Summarizing the above analyzed data, it appears obvious that metastatic melanoma patients included in clinical trials do not represent the whole patient population in real-world settings, due to exclusion criteria (e.g., brain metastases, rare mutations) and other factors (e.g., patient’s performance status) [23].

In 2013, targeted therapy of patients with melanoma was initiated in Russia. Vemurafenib and dabrafenib were the first of the new class of drugs registered in October 2013 (4 October 2013 for Vem and 10 October 2013 for Dabra). The next few drugs registered were the following—trametinib (6 April 2015); cobimetinib (17 February 2016) as a supplement to vemurafenib; ipilimumab, which was used as a second-line treatment in patients with advanced/metastatic melanoma (5 May 2016); pembrolizumab (18 November 2016); and nivolumab (22 December 2016); however, some drugs, such as encorafenib and binimetinib, have not yet been registered in the Russian Federation. Over the last decade, various clinical studies involving these registered drugs have been completed in this country.

Thus, the purpose of this study was to assess clinical outcomes in patients with BRAF V600 mutation-positive advanced melanoma who received BRAF-targeted therapy in real-world settings in large state medical centers in Russia (outside of clinical trials) and to evaluate treatment choices in routine clinical practice.

## 2. Materials and Methods

### 2.1. Patient Selection

This multicenter study, referred to as ADMIRE (Advanced Melanoma In Russia (Experience)), was an observational retrospective chart review study, conducted in a subset of patients with V600 BRAF-mutated unresectable or metastatic melanoma, who received targeted therapy in a real-world setting (NCT03663647). The study was conducted according to the guidelines of the Declaration of Helsinki and was approved by the Ethics Committee of the FSBI N. N. Blokhin National Medical Research Center of Oncology (NCT03663647). The study meets the requirements of the International Conference on the Harmonization of Good Clinical Practice guidelines applicable to observational research, patient privacy requirements, and ethical principles. Informed consent was obtained from all subjects involved in the study. The study did not aim to prove any formal hypothesis, it did not interfere with the medical care provided, and all medical events recorded during the study had already occurred or ended.

The data were collected from twelve medical centers from different federal regions in Russia. Each medical center is a large public cancer center/dispensary in its region. These include the N. N. Blokhin NMRC of oncology in Moscow and the N. N. Petrov NMRC of oncology in St. Petersburg, which are the largest federal budgetary research centers providing medical care for cancer patients from all Russian regions and are reference centers as well. Therefore, the patients enrolled in the study and the obtained data represent the real situation across the country. The analysis was expected to include no less than 300 medical records. The observational study required no intervention or interference with standard medical care; thus, it did not affect patient treatment. Key eligibility criteria were BRAF V600 mutation-positive unresectable or metastatic melanoma; age of ≥ 18 years at the start of treatment; targeted therapy for at least 2 months; and the minimum of one response assessment result.

### 2.2. Study Objectives

The primary objective was to evaluate the clinical outcomes of patients with V600 BRAF-mutant unresectable or metastatic melanoma who received TT in a real-world setting, assessed by line of therapy (first vs. second vs. later), LDH levels, brain metastases (mts), the subtype of BRAF V600 mutation, age, gender, and surgery at the advanced stage. The secondary objectives were to describe treatment patterns (percentage of TT, IT, and chemotherapy received in each line of treatment), safety profile, and finally to assess ORR, PFS, and OS. Outcome data from patient medical records were collected with the aim of providing real-world data on treatment patterns and duration, safety, best overall response rate, disease control rate (ORR + SD (stable disease), PFS, and OS). The objectives of the study were addressed in the overall population and in subgroups of clinical interest (age, gender, brain metastasis status, LDH level, type of BRAF V600 mutation, and surgery at the advanced stage).

The level of serum lactate dehydrogenase (LDH) is a recognized prognostic factor in malignant melanoma. We evaluated the LDH baseline level according to the laboratory test results obtained within 3 months prior to the commencement of TT. Patients were divided into 4 subgroups in accordance with the initial LDH level: (a) normal LDH level; (b) LDH up to 2 × the upper limit of normal (ULN); (c) LDH > 2 − 4 × ULN; (d) LDH > 4 × ULN.

### 2.3. Assessment Parameters

Tumor assessment was performed by the treating clinicians as per standard clinical practice (except for CT data, which was replaced by ultrasound analysis in some cases), rather than response evaluation criteria in solid tumors, and responses were documented as complete response (CR), partial response (PR), stable disease (SD), or progressive disease (PD).

Data sources included medical records, patients’ charts, and other related medical documentation that allowed the collection of information specified in the study variables. Evaluation of the eligibility criteria was performed by investigators before the start of data collection. Individual registration numbers for the study were assigned to each patient, and then all study variables were entered into the e-case report form (CRF). Adverse events (AEs) and serious adverse events (SAEs) were coded according to MedDRA. AEs/SAEs were characterized by numbers and frequencies.

### 2.4. Statistical Analysis

The primary objectives of the study were to evaluate clinical outcomes, such as progression-free survival (PFS), overall survival (OS), overall response rate (ORR), and disease control rate (DCR). PFS and OS were measured from the first drug administration to disease progression according to the local practice assessment, death, or last documented/reported visit. Patients who were alive at the end of the study were examined on the date of the last follow-up. The Kaplan–Meier method was used to calculate PFS and OS survival curves, and the log-rank test was used to compare these results. The multivariate analysis, performed with the Cox proportional hazards model, was used to evaluate correlations between various covariates (patients and treatment characteristics) as well as PFS and OS. Covariates included TT subtype (BRAFi vs. BRAFi + MEKi), age, gender, brain metastasis status, LDH level, type of subsequent therapy (IT vs. no IT). The hazard ratio (HR) and the 95% confidence interval (CI) for the HR were calculated for each factor. The differences were considered statistically significant if *p*-values were < 0.05.

Descriptive statistics was presented as percentages of total values for categorical variables and as medians for continuous and ordinal variables. Analysis of variance was used for the comparison of quantitative parameters between groups.

Intergroup comparisons of categorical data were performed using the Chi-squared test or, if necessary, Fisher’s exact test (if the expected frequency was less than 5 in any cell).

## 3. Results

The observed population included 382 patients across 12 centers in Russia. The date of the first enrollment after all approvals was 8 October 2018, and the date of the last completed register was 27 April 2019.

The mean age of the patients was 49.0 ± 13.4 years (18 years old (y.o.) to 82 years old). Patients were divided into two subgroups depending on the type of targeted therapy: BRAF inhibitor monotherapy (BRAFi) or combination therapy with BRAFi and MEKi. The patient population included 171 (44.7%) men and 211 (55.3%) women. There were no statistically significant differences between the treatment groups based on gender *(p* = 0.612) or age *(p* = 0.707).

ECOG status was registered for 316 patients of 382. ECOG patient status was as follows: ECOG 0-94 (29.8%), 1-176 (55.7%), 2-32 (10.1%), 3-12 (3.8%), 4-2 (0.6%), total-316 (100.0%) patients. Thus, 14 patients were at ECOG 3–4, and 32 patients were at ECOG 2, which often meets the criteria for the non-enrollment of such patients in clinical trials, whereas in our study these patients received antitumor therapy as well.

The characteristics of patients in each subgroup are presented in Table 1. The most common BRAF V600 subtype mutation was V600E (89.9%); other rare mutation subtypes (V600K, V600D, V600R, and others) were included in the study as well. The therapy types of all 382 patients included in the study were as follows: 44.8% *(n =* 171) of patients received BRAFi monotherapy and 55.2% *(n =* 211) received a combination of BRAFi and MEKi. The study registered the percentage of the therapy performed as follows: 59% of patients received TT as the first-line therapy, 23% as the second line, and 18% as the third or higher line. About half (52.6%) of the patients had a brain metastasis when TT was initiated.

Most patients (61.3%) received combined TT (dabrafenib (D) plus trametinib (T) or vemurafenib (V) plus cobimetinib (C)) as the first-line therapy, 55.7% as the second line, and 34.8% as the third or higher line. The ORR was higher in the combination group (BRAFi plus MEKi): 59.4%, 55.1%, and 50% for the first line, second line, and third or higher line, respectively, compared to BRAFi monotherapy (about 40% regardless of line). Analysis of the data on the ORR to targeted therapy depending on the subtype of TT (BRAFi monotherapy vs. BRAFi + MEKi combination) showed the best rates for patients undergoing combination treatment *(p* < 0.001). No statistically significant differences in ORR were found *(p* > 0.05) regarding the TT line. Of note, the largest and most statistically significant differences in response between the monotherapy and combination groups were observed in patients receiving the first-line therapy (*p* = 0.003).

**Figure 1:**
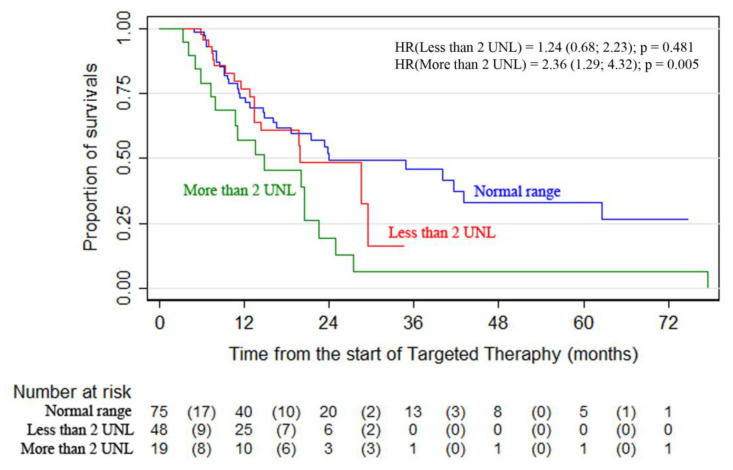
Targeted therapy results according to the initial LDH levels *(p* = 0.0156).

Statistically significant differences were revealed between the overall survival curves in the subgroups of patients arranged according to LDH levels. The highest survival was observed in patients with normal LDH levels—24-month OS was 51.8% (95% CI, 37.3–64.5) vs. 19.6% (95% CI, 5.0–41.1) for patients with LDH > 2ULN *(p* = 0.0156) (Figure 1, Table 2). The analysis showed that the combination therapy subgroup had more patients with elevated LDH levels. Surgery as part of advanced melanoma therapy was performed on 107 patients (28%). It was most commonly performed during the first-line therapy (60 patients).

**Figure 2:**
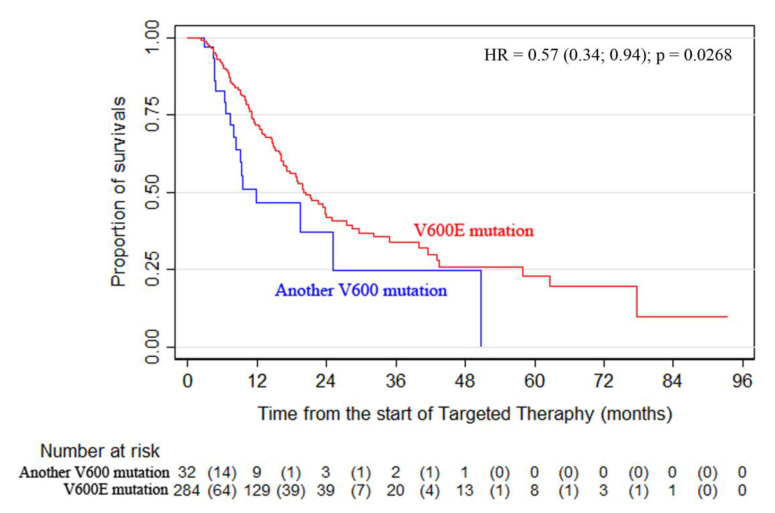
Overall survival according to the mutation subtype *(p* = 0.0268).

The analysis of BRAF V600 subtype data showed that mutation V600E was detected in 284 patients (89.9%), whereas 32 patients had mutations of a different subtype (D, K, R, etc.). Comparison of OS curves using a log-rank test revealed statistically significant differences between patients with the V600E mutation and the rare subtype mutations (D, K, R, etc.): mOS of 20.5 months vs. 11.9 months, respectively, and 12- and 24-month OS rates of 71.8% vs. 46% and 43.0% vs. 36.8%, respectively *(p* = 0.0268) (Figure 2). Similar results were observed for the PFS data *(p* = 0.0481).

Statistically significant factors in the OS analysis of the first-line patients were gender *(p* = 0.010), the presence of brain metastases *(p* = 0.018), and IT after TT *(p* = 0.001); there was also a tendency towards a statistically significant association with LDH levels *(p* = 0.063). Other factors showed no statistically significant association with the OS of the first-line patients *(p* > 0.05).

The risk of OS in patients receiving the second-line combination therapy with BRAFi + MEKi was almost half of that compared with patients on monotherapy, HR = 0.485 *(p* = 0.159). The statistically significant factors of the model included surgical treatment of advanced melanoma *(p* = 0.044), LDH level *(p* = 0.012), the number of organs with metastases (*p* = 0.003), and IT after TT (*p* < 0.05).

Surgical treatment of metastatic melanoma dramatically reduced the risk of death from all causes with HR = 0.338, i.e. three times on average. However, the magnitude of the effect for various factors of the model was highly uncertain due to the small sample size. Other factors had no statistically significant association with OS *(p* > 0.05).

The risk of death from all causes for the patients receiving third- or higher-line therapy (BRAFi + MEKi therapy) was reduced by almost 25% compared with that for patients on BRAFi monotherapy, HR = 0.571; however, statistical significance was not achieved *(p* = 0.537). None of the studied factors had a statistically significant association with OS. A trend was noted for the factors of availability of surgical treatment and IT *(p* = 0.106 and *p* = 0.092, respectively).

Brain metastases were registered in 201 (52.6%) of the total patients included in the study. The data analysis demonstrated that the median OS (mOS) was 16.8 months from the first metastasis (95% CI, 14.4–21.5); 24-month OS was 37.6%. Median PFS accounted for 11.2 and 24-month PFS was 16% (95% CI, 10.2–22.9).

**Figure 3:**
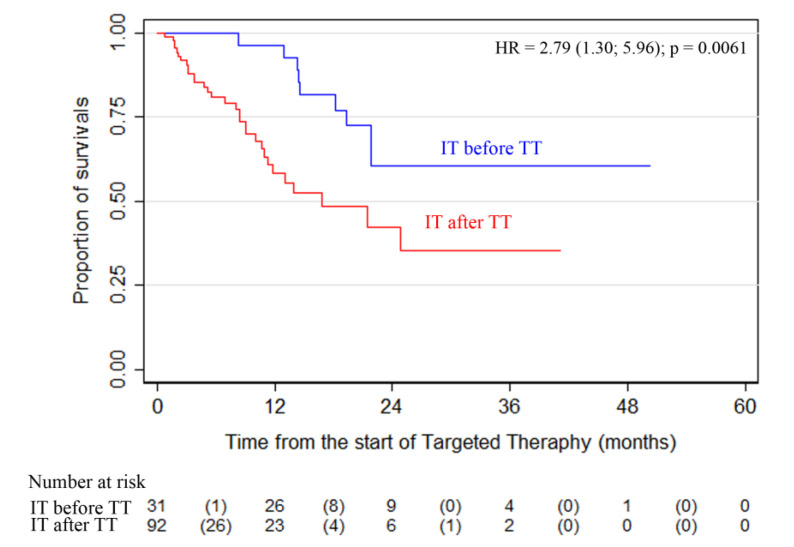
Overall survival in patients who received immunotherapy with anti-PD1 agents before or after targeted therapy *(p* = 0.0061).

Another important finding was that patients who received IT (anti-PD1) before TT demonstrated a significantly higher survival rate. Comparison of the overall survival of patients who received IT (anti-PD1) before *(n =* 31) or after TT *(n =* 92) showed that the survival rate in the subgroup of patients with IT before TT was significantly higher than in the patients who started anti-PD1 IT after TT (Figure 3; *p* = 0.0061).

Considering safety results, the study did not present any new data. The records showed 418 AEs in 147 patients (38.4%). The most common AEs were associated with general disorders (140 episodes in 79 patients), as well as skin and subcutaneous tissue disorders (123 episodes in 82 patients). Pyrexia was the most common (56 episodes in 45 patients) of all general disorders, whereas fatigue was observed in 29 patients. The most frequent skin disorders were skin rash (in 40 patients) and dry skin (in 17 patients).

In addition, the registered AEs included a grade 5 event in one patient (a fatal stroke in patient 10013CC), grade 4 events in nine patients (2.4%), grade 3 AEs in 40 patients (10.4%), two events in 47 patients (12.3%), and grade 1 AEs associated with all described treatment patterns in 45 patients (11.8%).

## 4. Discussion

This study reports the survival, efficacy and safety data obtained in 382 patients with advanced BRAF V600 mutant melanoma who received TT in a real-world setting in Russia. To date, this is the largest analyzed cohort of patients who received TT outside clinical trials. The results of the retrospective analysis demonstrated that TT was well tolerated and controlled in patients with BRAF V600 mutation-positive unresectable or metastatic melanoma in a real-world setting. The study results of ORR, PFS, and OS confirmed the significant clinical effectiveness of TT in a real-world setting in the treatment of patients with advanced disease who had poorer prognostic factors compared to patients enrolled in phase 3 clinical trials. For instance, patients with brain metastasis are generally excluded from prospective phase 3 clinical trials, whereas in the given study 52.6% of patients had a brain metastasis at the initiation of TT. The ADMIRE study showed that patients with brain metastases (who had or had not undergone prior local therapy for brain metastases) demonstrated mPFS and mOS of 11.2 months (95% CI, 10.0–12.5) and 16.8 months (95% CI, 14.4–21.5), respectively, estimated from the diagnosis of the first metastasis.

The comparison of the results of phase III randomized clinical trials (coBRIM, COMBI-v/d) and the real-word ADMIRE study clearly demonstrates that patients from real practice have more unfavorable characteristics: the presence of brain mutations, ECOG performance status 2–4, and rare types of BRAF mutations. However, we observed a high efficacy of targeted therapy in routine practice despite more frequent unfavorable events. Thus, the median PFS in the ADMIRE study for the patients receiving combined first line therapy was 9.2 months versus 11.1 months in the pooled COMBI-v/d data analysis (dabrafenib + trametinib), 12.3 months in the coBRIM study (vemurafenib + cobimetinib), and 14.9 months in the COLUMBUS study (encorafenib + binimetinib). The median OS in ADMIRE for the first-line combined TT group was 22.6 months versus 25.9 months in the pooled COMBI-v/d data analysis (dabrafenib + trametinib), and 22.5 months in the coBRIM study (vemurafenib + cobimetinib); whereas the 24-month OS was 47.2% vs. 52% and 49%, respectively. It should be noted that these RCTs included patients with BRAF V600E/K mutations, with no brain metastases, and with an ECOG PS of less than two.

In addition, the ADMIRE analysis showed that overall survival was better in those patients who had surgery for metastases at the advanced stage compared to patients who had no surgical treatment. The safety and effectiveness outcomes observed in the study were comparable and consistent with early data reported from controlled clinical trials. The most commonly reported AEs were pyrexia for the D + T regimen and rash and fatigue for the V + C regimen. No new AEs were observed. As expected, the frequency of AEs was lower than in randomized clinical trials.

The high efficacy (ORR) of targeted therapy achieved in phase 3 studies was confirmed by our study in patients receiving the first-line therapy in routine practice. It is important to note that the present study also showed high efficacy in the second and subsequent lines of treatment, whereas phase 3 RCTs studied the efficacy of the first line of therapy only. The PFS and OS rates were lower during the third- or higher-line therapy, but better than historical data. Additional analysis of treatment sequences revealed that the subgroup of patients who received IT before TT had higher survival rates in the ADMIRE study. It should be noted, however, that the sample size was not balanced in the groups comparing OS in patients receiving IT before TT or IT after TT (31 vs. 92). The literature reports of the completed studies showed that immunotherapy before targeted therapy could provide more favorable results even in patients with *BRAF* mutations [24,25,26,27,28]. Considering the results of our analysis, the better prognosis in the IT group before TT could be attributed to a lower number of patients receiving IT or a larger number of patients with a “good” prognosis who were prescribed anti-PD1 immunotherapy before TT. Nevertheless, this sequence of the treatment requires further research and observation.

Atkinson et al. conducted a retrospective study, DESCRIBE II, consisting of a chart review of patients with BRAF V600-mutated unresectable stage III/IV melanoma receiving dabrafenib plus trametinib as compassionate use. Treatment patterns and duration, clinical outcomes, and tolerability were evaluated (Table 3). The total number of enrolled patients was 271; 92.6% of them had stage IV melanoma, including 36.5% with brain metastases. One hundred sixty-two patients (59.8%) were BRAFi naive. One hundred seventy-one patients (63.1%) received first-line dabrafenib plus trametinib. The BRAFi-naive patients achieved the overall response rate (ORR) in 67.3% cases, median OS (mOS) reached 20.0 months, and median progression-free survival (mPFS) was 7.5 months. The number of BRAFi-naive patients with detected brain metastases was 62, ORR was 61.3%, mOS was 15.5 months, and mPFS was 6.2 months [29]. DESCRIBE II was similar to the ADMIRE study in terms of patient characteristics: 36.5% had brain metastases, two patients had ECOG 3 and 16 patients ECOG 2, and 10 patients had other types of BRAF mutations. However, DESCRIBE II had no data on the variants of other BRAF mutations and their impact on the therapy results. ADMIRE, on the other hand, presents data about 382 patients (vs. 271), including 32 patients with rare types of mutations (K, D, R, and others) and provides the analysis of their impact on PFS and OS (described above in the Results section).

Another study using real-world data was performed in Poland. A retrospective analysis included 287 patients with unresectable stage III and stage IV melanoma treated at the Maria Sklodowska-Curie National Research Institute of Oncology, Krakow Branch, from 2013 to 2019. All enrolled patients were treated with immunotherapy (IT; consisting of pembrolizumab/nivolumab or ipilimumab) or targeted therapy (TT; consisting of vemurafenib ±cobimetinib or dabrafenib ±trametinib) in at least one treatment line. BRAF mutations were detected in 152 (55%) patients. In general, the majority of patients (92%) showed a very good or good performance status (Eastern Cooperative Oncology Group (ECOG) 0 or 1). Brain metastases were detected in 64 (22%) patients. Median OS and PFS in the experimental group from the start of the first-line treatment were 14.9 and 6.7 months, respectively. The first-line treatment of patients involved IT, TT, or chemotherapy, and the median OS reached 19.2, 12.6, and 15.9 months, respectively [30]. Thus, the few available studies with real-world data showed positive achievements of targeted therapy for the cohort of BRAF-mutant melanoma patients. Moreover, our study of real-world clinical practice was the largest and included 2.5-fold more patients with BRAF-mutant advanced melanoma compared to those of the above-mentioned study (382 vs. 152 patients) and demonstrated increased median OS (22.6 months) and 2-year OS (47.2%) compared to the earlier reported study [30] in patients receiving the first-line targeted therapy. In addition, even though the patients included in the ADMIRE study had poorer prognostic factors, the data showed essentially higher effectiveness in a real-world setting, which may correspond to phase 3 clinical trials.

However, it is necessary to take into account the fact that retrospective chart reviews are subject to inherent methodological and operational limitations, including incomplete data, the observational period limiting the total amount of available follow-up data, and possible underreporting of AEs. Unlike controlled clinical trials, tumor assessments were not necessarily performed according to the response evaluation criteria in solid tumors (RECIST) but based on the clinical judgement of the treating physician as per clinical practice.

## 5. Conclusions

The ADMIRE study demonstrated that targeted therapy showed high effectiveness in routine clinical practice, in particular in patients with unfavorable prognostic factors, such as brain metastases, which were detected in over half of patients (52.6%) at the initiation of TT. Additionally, efficacy analysis of the sequential therapy showed that patients who received anti-PD1 IT prior to TT achieved a significantly higher survival rate. Another important finding is that patients with more than one line of treatment had a better prognosis. Notably, the median PFS in the second and third lines was similar.

The study revealed a clear difference in the treatment outcomes for patients with the V600E mutation and rare subtype mutations (D, K, R, etc.)—mOS of 20.5 months vs. 11.9 months, respectively, and 12- and 24-month OS rates of 71.8% vs. 46% and 43.0% vs. 36.8%, respectively *(p* = 0.0268). Furthermore, the study found that surgical treatment of metastatic melanoma dramatically reduced the risk of death from all causes (HR = 0.338).

Therefore, the use of targeted therapy beyond clinical trials significantly improves the survival of patients in a real-world setting, including patients with brain metastases and rare types of BRAF mutation.

The approval of the new drugs for targeted therapy and immune therapy led to a significant increase in the progression-free and overall survival of real-life patients with metastatic melanoma.

Evidence generated from real-world data makes an important contribution to the evidence obtained from clinical trials in thoroughly selected patients, and may be transferred to a broader patient population. Further studies should be conducted to assess the effectiveness of various treatment sequences.

## Figures and Tables

**Table 1:** Patient characteristics and results.

Parameters		All Patients, n (%)	Type of Targeted Therapy	
			BRAFi	BRAFi + MEKi
Median age, years		49.0 ± 13.4 years (18 y.o. to 82 y.o.)	49.3 ± 13.2 years (18.6 y.o. to 79.9 y.o.)	48.7 ± 13.5 years (19.8 y.o. to 82 y.o.)
Gender	Male	171/382 (44.7%)	79/171 (46.2%)	92/171 (53.8%)
	Female	211/382 (55.3%)	92/211 (43.6%)	119/211 (56.4%)
Stage at targeted therapy initiation according to TNM 8	N1-3M0 (III unresectable)	17 (4.5%)	6 (2.9%)	11 (6.4%)
	M1 (unknown)	5 (1.3%)	3 (1.5%)	2 (1.2%)
	M1a	54 (14.3%)	26 (12.7%)	28 (16.3%)
	M1b	46 (12.2%)	39 (19.0%)	7 (4.1%)
	M1c	136 (36.1%)	82 (40.0%)	54 (31.4%)
	M1d	119 (31.6%)	49 (23.9%)	70 (40.7%)
	All available data	377 (100.0%)	205 (100.0%)	172 (100.0%)
Variant of V600 BRAF mutation, n (%)	E	284 (89.9%)	125 (88.7%)	159 (90.9%)
	K	23 (7.3%)	12 (8.5%)	11 (6.3%)
	D	2 (0.6%)	1 (0.7%)	1 (0.6%)
	R	3 (0.9%)	1 (0.7%)	2 (1.1%)
	Other	4 (1.3%)	2 (1.4%)	2 (1.1%)
	All available data for mut type	316 (100.0%)	141 (100.0%)	175 (100.0%)
	V600 mutation unknown subtype	66		
Treatment line in which targeted therapy was used, n (%)	First line	225 (59%)	87/225 (38.7%)	138/225 (61.3%)
	Second line	88 (23%)	39/88 (44.3%)	49 (55.7%)
	Third line or higher	69 (18%)	45 (65.2%)	24 (34.8%)
	All data	382 (100%)	171 (44.8%)	211 (55.2%)
Brain metastasis, n (%)	First line	111/225 (49.3%)	38/87 (43.7%)	73/138 (52.9%)
	Second line	54/88 (61.4%)	20/39 (51.3%)	34/49 (69.4%)
	Third line or higher	36/69 (52.2%)	25/45 (55.6%)	11/24 (45.8%)
	All data	201/382 (52.6%)	83/171 (48.5%)	118/211 (55.9%)
LDH level, n (%)	Unknown data	240/382 (62.8%)		
	First line	79:	21:	58:
	(a) N	48	14	26
	(b) N-2UNL	28	4	24
	(c) 2-4UNL	8	2	6
	(d) >4UNL	3	1	2
	Second line	36:	16:	20:
	(a) N	22	12	10
	(b) N-2UNL	12	4	8
	(c) 2-4UNL	2	0	2
	(d) >4UNL	0	0	0
	Third line or higher	27:	16:	11:
	(a) N	13	5	8
	(b) N-2UNL	8	6	2
	(c) 2-4UNL	2	2	0
	(d) >4UNL	4	3	1
	All available data	142/382 (37.2%)	53/142	89/142
Surgery at advanced stage, n (%)	First line	60 (56%)	27 (25.2%)	33 (30.8%)
	Second line	25 (23.4%)	8 (7.5%)	17 (15.9%)
	Third line or higher	22 (20.6%)	14 (13.1%)	8 (7.5%)
	All data	107/382 (28%)	49/107 (45.8%)	58/107 (54.2%)
Objective response rate (ORR), CR + PR, n (%)	First line	116/225 (51.6%)	34/87 (39.1%)	82/138 (59.4%)
	Second line	43/88 (48.9%)	16/39 (41.0%)	27/49 (55.1%)
	Third line or higher	30/69 (43.5%)	18/45 (40%)	12/24 (50%)
	All lines	189/382 (49.5%)	68/171 (39.8%)	121/211 (57.4%)
Disease control rate, n (%)	First line	74.7%	65.5%	80.4%
	Second line	75%	74.4%	75.5%
	Third line or higher	72.5%	71.1%	75%
	All lines	74.3%	69%	78.7%

**Table 2:** Overall survival rates of patients with different LDH baseline analyzed on the base of Kaplan–Meier curves.

Subgroup LDH Level	N	Overall Survival, CI 95%		HR (CI 95%)
		Median OS, Months	24-Month OS, %	
Normal range	75	24.0 (16.1–43.0)	51.8 (37.3–64.5)	Ref.
Less than 2 UNL	48	19.9 (13.5–29.6)	49.8 (29.6–67.0)	1.24 (0.68; 2.223)
More than 2 UNL	19	14.8 (7.2–20.5)	19.6 (5.0–41.1)	2.36 (1.29; 4.32)

**Table 3:** Indirect comparison between phase 3 trial results and real-world experience; data from different studies.

	COMBI-d/v [2]	coBRIM	Real-World Experience (ADMIRE)			DESCRIBE II (Named Patient Program) [29]		
Drugs	D+T	V+C	D+T and V+C	D+T and V+C	D+T and V+C	D+T	D+T	D+T
Line of therapy	first	first	first	second	third or higher	first	second	third or higher
N	563	247	138/225	49/88	24/69	140	17	5
ORR, %	68	70	59.4	55.1	50	66.4	72.2	
Median PFS (95% CI), months	11.1 (9.5–12.9)	12.3 (9.5–13.4)	9.2 (8.1–10.1)	9.4 (5.5–13,1)	7.4 (4.7–9.1)	8.0	7.1	
						7.5 (6.3–9.3)		
24-month PFS, (95% CI), %	31% (27–35)	32%	17.1 (9.9–26.1)	19.0 (5.1–39.4)	13.2 (2.7–32.2)	0		
Median OS (95% CI), months	25.9 (22.6–31.5)	22.5 (20.3–28.8)	22.6 (19.0–34.9)	16.1 (14.8–ND) (not defined)	17.1 (9.9–ND)	20.0	15.1	
24 month OS, %	52% (48–57)	49%	47.2% (34.1–59.2)	43.4% (22.2–63.0)	20.6% (1.4–55.4)			
